# Editorial: Pediatric obesity: how to diverge from developmental pathways?

**DOI:** 10.3389/fendo.2024.1363099

**Published:** 2024-01-23

**Authors:** Alexandra Soldatou, Anastasia Garoufi

**Affiliations:** ^1^ 2^nd^ Department of Pediatrics, National and Kapodistrian University of Athens, School of Medicine, Athens, Greece; ^2^ National and Kapodistrian University of Athens, School of Medicine, Athens, Greece

**Keywords:** lifestyle intervention, nonalcoholic fatty liver disease - NAFLD, dysglycemia, overweight, childhood

It is well established that obesity in childhood tracks into adulthood. Clinical and subclinical complications of obesity may occur as early as infancy, conferring an increased risk of disease and precocious death (1). Unfortunately, the lifetime course of obesity is notoriously hard to change, unless key modifiable factors are identified, and collaborative solutions are developed (2, 3). Therefore, if we focus on prevention strategies, the obesity pathway may not be inevitable (4).

In this Research Topic, five articles, one systematic review and meta-analysis and four original articles, explore diagnostic and predictive biomarkers in main complications of pediatric obesity, treatment strategies and transfer of evidence-based care models.

In a qualitative study by Sierra-Velez et al., barriers and facilitators for the implementation of Healthy Weight Clinic (HWC), an evidence-based pediatric weight management intervention (PWMI) developed jointly by the American Academy of Pediatrics and Massachusetts General Hospital, are described. Specifically, the researchers interviewed 20 stakeholders - 10 serving two health centers in Mississippi where the HWC will be piloted (pre-implementation sites), and 10 serving health centers in Massachusetts where the HWC had already been implemented (sites in maintenance stages). Interestingly, the themes that emerged were similar for both groups of stakeholders. Factors identified were categorized in five broad conceptual domains: characteristics of individuals, inner setting, outer setting, intervention characteristics, and process. Important findings relevant to the adaptation of evidence-based models in new settings include a positive learning climate at the health center, the engagement of highly motivated, culturally, and weight-sensitive staff who believe in the effectiveness of the intervention, seek creative solutions to overcome social barriers, and work collaboratively both within their multidisciplinary team and their community.

Due to the global obesity epidemic, a staggering increase of pediatric Non-Alcoholic Fatty Liver Disease (NAFLD) is observed; NAFLD is the most frequent pediatric chronic liver disease, recognized in increasingly younger children and associated with significant comorbidities. Since delayed diagnosis often results in advanced disease with the identification of fibrosis on liver biopsy, more, less invasive, and more reliable biomarkers are needed to screen obese children (5). The goal of the retrospective study by Zhou et al. was to find salient clinical characteristics and validate a prediction model for NAFLD. The study included 3,216 obese Chinese children and adolescents, classified in three groups: without NAFLD, with Non-Alcoholic Fatty Liver (NAFL) and with Non-Alcoholic Steatohepatitis (NASH). 3,036 participants served as the training set for the development of the NAFLD prediction model, and the remaining 180, who underwent Liver Hydrogen Proton Magnetic Resonance Spectroscopy (1H-MRS) as the validation set. Surprisingly, NAFLD was the most frequent complication of obesity, observed in 59.5% of children, predominantly boys and peaking at 10 – 12 years of age. The main comorbidity of NAFLD was hyperuricemia (59.6%), followed by dyslipidemia, hypertension, metabolic syndrome and dysglycemia. The cardiometabolic marker found with the closest association with pediatric NAFLD was TyG-WC [(Triglyceride – glucose index) – (waist circumference)]. Based on the data collected, the researchers developed a prediction model with eight anthropometric and laboratory parameters. The model’ s sensitivity and specificity proved satisfactory with AU-ROC of 0.821. In addition, liver 1H-MRS was used to validate the model. Of note, the quantification of fat in NAFLD based on 1H-MRS results is comparable with that of liver biopsy. Thus, the research by Zhou et al. has contributed significantly to the improved detection, assessment, and follow-up of pediatric NAFLD in obese children.

In their review and metanalysis, Li et al. investigated the effectiveness of lifestyle interventions in the management of overweight/obesity in Chinese children and adolescents, 6 – 18 years old. Following search of five databases, the researchers found eight Randomized Controlled Trials (RCTs) of dietary, physical exercise and education health interventions implemented in an aggregated number of 425 overweight/obese children for a period of 2 months to 2 years. Comparisons were conducted with a control group of 420 children. The primary outcome was the change of BMI, and secondary outcomes were changes in metabolic parameters, such as fasting glucose and lipid profile, and changes in blood pressure measurements. All studies examined showed that lifestyle interventions resulted in significant decrease of BMI in overweight/obese children compared to the control group. Effectiveness was even higher in multiple-component and longer term (> 1 year) interventions, and those targeting adolescents. Significant changes in fasting glucose, lipid profile and blood pressure were noted in one, three and two studies respectively. The researchers’ findings may be useful to develop more effective lifestyle interventions to manage pediatric overweight/obesity in China.

Parajuli et al. studied 108 youth (4 – 21 years old) with prediabetes on oral metformin treatment retrospectively to determine the impact of adherence with a regular nutrition visit plan (four visits/year) to a registered dietician on HbA1c levels and the progression to Type 2 Diabetes (T2D). Within four years of follow up, 17% of the total population developed T2D; 9.1% of youth in the adherent group (≤ 2 visits/year) and 22.6% of those in the non-adherent group (¾ 1 visit/year). In addition, the mean time of progression of prediabetes to T2D was significantly lower in the non-adherent group. Following control of confounding factors, a significant difference in HbA1c trend was found between the two groups. HbA1c was initially higher in the adherent group, decreased significantly and remained lower than that of the non-adherent group in the 4-year follow-up. In the contrary, there was no significant change in the BMI z-score observed. This suggests the decrease in HbA1c was independent of changes in BMI, supporting the notion that glycemic changes may herald anthropometric and may be interpreted by the decrease in insulin resistance markers. This study concluded that adherence with nutrition visits is very important, resulting in a four-fold decreased risk and lengthening of period of progression of prediabetes to T2D.

Based on encouraging findings in adults (6), Naguib et al. explored the feasibility and effectiveness of continuous glucose monitoring (CGM) in 43 adolescents with obesity, without diabetes, who participated in a 12-week trial of time restricted eating (TRE). With the use of a wearable device, CGM provided noninvasive glucose measurements that identified glycemic excursions during fasting and non-fasting periods, suggesting potential application in monitoring adherence to TRE along with self-report of dietary intake. In addition, CGM was well tolerated and accepted in this group of adolescents. Although glycemic profiles were similar in adolescents practicing TRE and controls, the researchers suggest the impact of TRE on glycemic variability merits further investigation with appropriately powered studies. Therefore, new, interesting uses of CGM in adolescents with obesity are suggested in the study by Naguib et al.

In conclusion, in this Research Topic five important pathways to halt the progression of childhood obesity and its complications are highlighted. First, the implementation of evidence-based weight management interventions in new settings calls for positive attitudes and creativity among healthcare staff, workplaces, and communities. Second, a model with eight anthropometric and laboratory parameters can predict NAFLD in obese children, signifying new opportunities for timely and non-invasive diagnosis. Third, multi-dimension lifestyle interventions lasting over one year and targeting adolescents hold promise for the management of overweight/obesity and its complications. Fourth, adherence of youth with prediabetes to a regular nutrition visit plan may improve HbA1c levels, decrease the risk and delay the progression to diabetes. Fifth, CGM may be a useful adjuvant in the monitoring of glucose and adherence to TRE in adolescents with obesity. We hope the findings of our Research Topic inspire clinicians and researchers for future actions to combat the global obesity epidemic in childhood with significant implications for the health of the next generation of adults.

## Author contributions

AS: Writing – original draft. AG: Writing – review & editing.
